# The Splicing Factor Proline-Glutamine Rich (SFPQ/PSF) Is Involved in Influenza Virus Transcription

**DOI:** 10.1371/journal.ppat.1002397

**Published:** 2011-11-17

**Authors:** Sara Landeras-Bueno, Núria Jorba, Maite Pérez-Cidoncha, Juan Ortín

**Affiliations:** 1 Centro Nacional de Biotecnología (CSIC), Campus de Cantoblanco, Madrid, Spain; 2 CIBER de Enfermedades Respiratorias, ISCIII, Bunyola, Mallorca, Spain; Johns Hopkins University - Bloomberg School of Public Health, United States of America

## Abstract

The influenza A virus RNA polymerase is a heterotrimeric complex responsible for viral genome transcription and replication in the nucleus of infected cells. We recently carried out a proteomic analysis of purified polymerase expressed in human cells and identified a number of polymerase-associated cellular proteins. Here we characterise the role of one such host factors, SFPQ/PSF, during virus infection. Down-regulation of SFPQ/PSF by silencing with two independent siRNAs reduced the virus yield by 2–5 log in low-multiplicity infections, while the replication of unrelated viruses as VSV or Adenovirus was almost unaffected. As the SFPQ/PSF protein is frequently associated to NonO/p54, we tested the potential implication of the latter in influenza virus replication. However, down-regulation of NonO/p54 by silencing with two independent siRNAs did not affect virus yields. Down-regulation of SFPQ/PSF by siRNA silencing led to a reduction and delay of influenza virus gene expression. Immunofluorescence analyses showed a good correlation between SFPQ/PSF and NP levels in infected cells. Analysis of virus RNA accumulation in silenced cells showed that production of mRNA, cRNA and vRNA is reduced by more than 5-fold but splicing is not affected. Likewise, the accumulation of viral mRNA in cicloheximide-treated cells was reduced by 3-fold. In contrast, down-regulation of SFPQ/PSF in a recombinant virus replicon system indicated that, while the accumulation of viral mRNA is reduced by 5-fold, vRNA levels are slightly increased. *In vitro* transcription of recombinant RNPs generated in SFPQ/PSF-silenced cells indicated a 4–5-fold reduction in polyadenylation but no alteration in cap snatching. These results indicate that SFPQ/PSF is a host factor essential for influenza virus transcription that increases the efficiency of viral mRNA polyadenylation and open the possibility to develop new antivirals targeting the accumulation of primary transcripts, a very early step during infection.

## Authors Summary

The influenza A viruses cause annual epidemics and occasional pandemics of respiratory infections that may be life threatening. The viral genome contains 8 RNA molecules forming ribonucleoproteins that replicate and transcribe in the nucleus of infected cells. Influenza viruses are intracellular parasites that need the host cell machinery to replicate. To better understand this virus-cell interplay we purified the viral RNA polymerase expressed in human cells and identified several specifically associated cellular proteins. Here we characterise the role of one of them, the proline-glutamine rich splicing factor (SFPQ/PSF). Down-regulation of SFPQ/PSF indicated that it is essential for virus multiplication. Specifically, the accumulation of messenger and genomic virus-specific RNAs was reduced by SFPQ/PSF silencing in infected cells. Furthermore, transcription of parental ribonucleoproteins was affected by SFPQ/PSF down-regulation. The consequences of silencing SFPQ/PSF on the transcription and replication of a viral recombinant replicon indicated that it is required for virus transcription but not for virus RNA replication. In vitro transcription experiments indicated that SFPQ/PSF increases the efficiency of virus mRNA polyadenylation. This is the first description of a cellular factor essential for influenza virus transcription and opens the possibility to identify inhibitors that target this host-virus interaction and block virus gene expression.

## Introduction

The influenza A viruses belong to the family *Orthomyxoviridae* and contain a segmented, single-stranded RNA genome of negative polarity (for a review see [1]. Each of the genomic RNA segments is encapsidated in a ribonucleoprotein particle (RNP) containing the polymerase complex and a number of nucleoprotein (NP) monomers, depending on their size [2], [3]. Contrary to many other RNA viruses, the influenza virus RNPs are transcribed and replicated in the nucleus of infected cells. The enzyme responsible for these activities is the viral polymerase, a heterotrimer that comprises the PB1, PB2 and PA subunits [4]–[6]. The PB1 subunit acts as polymerase [7], [8] while PB2 and PA are responsible for cap-binding and cap-snatching, respectively [9]–[12]. The heterotrimer has a compact structure [2], [13]–[15] and is required for both transcription and replication [7], [16]–[19]. The polymerase complex can be found associated to the RNP structure or in a soluble form [20], the latter being able to oligomerise *in vivo* [21], [22]. Along the years, a number of human cell factors have been described as interactors of influenza virus polymerase and in some specific cases their role in virus replication has been studied [23]–[36].

In one such studies, we identified the human SFPQ/PSF factor as associated *in vivo* to influenza virus polymerase by proteomic analysis of purified complexes [34]. Human SFPQ/PSF is a nuclear multifunctional protein that has been implicated in a series of steps in the human gene expression pathway (for a review, see [37]. It was first described as associated to the polypyrimidine tract-binding protein (PTB) [38] and contains regions rich in arginine/glycine and proline/glutamine close to its N-terminus as well as two RRMs located more C-terminal. SFPQ/PSF can be found as a heterodimer with p54nrb/NonO, a protein that is highly homologous to the SFPQ/PSF C-terminal half. The SFPQ/PSF-p54nrb/NonO heterodimer co-purifies with DNA topoisomerase and interacts with RAD1 recombinase, leading to the stimulation of nucleic acid strand transfer and the cleavage/religation steps [39]–[43]. In addition, several reports have shown that SFPQ/PSF and/or p54nrb/NonO can regulate cellular transcription in a variety of genes (reviewed in [37]. In agreement with the SFPQ/PSF association with PTB, its binding has been reported to elements in the splicing machinery, like the U4/U6-U5 tri-snRNP and many other splicing factors [44]–[46] and the RNA pol II CTD [47], probably in a RRM-dependent manner [48]. Consistent with these interactions, SFPQ/PSF has been shown to stimulate the splicing of mRNAs transcribed by strong transcriptional activators and to control alternative splicing [49], [50]. In spite of the above mentioned associations, most of SFPQ/PSF-p54nrb/NonO can be found in the “nuclear matrix” fraction [51], consistent with the proposed role for the heterodimer in the specific retention in the nuclear matrix of RNA which has been A>I hyperedited [37], [52].

In addition to these many potential functions assigned to SFPQ/PSF or the SFPQ/PSF-p54nrb/NonO heterodimer, the former has been shown to bind specifically to a defined stem-loop in hepatitis delta RNA [53] and the 3′-end of HCV genome [54], while the latter inhibits the transport and expression of HIV mRNAs containing the instability region (INS) [55]. Here we have analysed the role of SFPQ/PSF in the influenza virus infection. Silencing the SFPQ/PSF gene, but not p54nrb/NonO, strongly reduced virus multiplication. The accumulation of viral genomic vRNA and mRNA as well as viral proteins was reduced, probably as a consequence of the inhibition of primary and secondary transcription, but normal splicing of virus mRNA was observed. *In vitro* transcription of recombinant RNPs generated in SFPQ-silenced cells resulted in reduced levels of viral poly A^+^ RNA. These results are consistent with a role for SFPQ/PSF during the polyadenylation step in the synthesis of viral mRNAs from the parental RNP templates, the earliest nuclear step in virus replication cycle, as well as during secondary transcription.

## Results/Discussion

**Figure 1:**
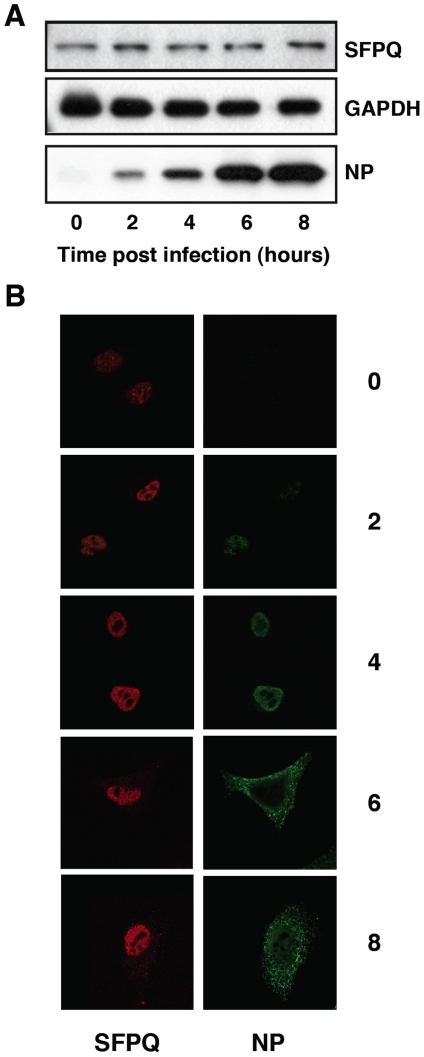
Accumulation and localisation of SFPQ/PSF in influenza virus infected cells. Cultures of human A549 cells were infected with the VIC strain of influenza virus at 3 pfu/cell. (A) At the times after infection indicated (hours), extracts were prepared and analysed by Western-blot with the antibodies indicated to the right. (B) At the same times, cultures were fixed and processed for immunofluorescence using antibodies specific for SFPQ/PSF and NP, as indicated.

A proteomic analysis of the intracellular complexes formed by recombinant influenza virus polymerase revealed a series of human proteins that were stably associated to the viral enzyme [34]. One of such associated factors was SFPQ/PSF, a multifunctional nuclear protein involved in transcription, post-transcriptional processing of mRNAs and DNA rearrangements [37]. To study the role of SFPQ/PSF in influenza virus replication we first analysed the expression and localisation of the protein along the infection cycle. Cultures of A549 cells were infected with VIC influenza virus at high multiplicity and total cell extracts were prepared at various times thereafter. The accumulation of SFPQ/PSF was determined by Western-blot using specific antibodies and is shown in Figure 1A. No changes in the level of expression of the protein were observed when compared to GAPDH as a standard, whereas the progressive accumulation of virus proteins was apparent (see NP marker in Figure 1A). Similar experiments were obtained when the WSN virus strain was used (data not shown). The localisation of SFPQ/PSF was analysed by confocal immunofluorescence and is presented in Figure 1B. The protein was found in a punctuate pattern within the nucleus, slightly more intense in the nuclear periphery and excluded from the nucleoli. No significant change could be observed in such distribution along virus infection, although a small increase in cytoplasmic staining was apparent at late times (Figure 1B). Similar results were obtained using the HeLa cell line and the WSN virus strain (data not shown).

### SFPQ/PSF is specifically required for efficient influenza virus infection

**Figure 2:**
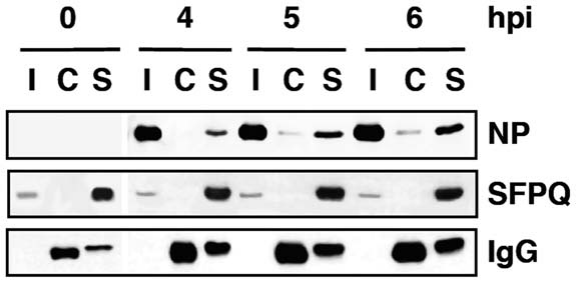
Association of viral ribonucleoproteins with SFPQ/PSF. Cultures of human A549 cells were infected with the VIC strain of influenza virus at 3 pfu/cell. Total cell extracts were prepared at the times after infection indicated (hpi) and immunoprecipitated with anti-SFPQ/PSF or control antibodies. The immunoprecipitates were analysed by Western-blot using antibodies specific for SFPQ/PSF or NP, as indicated. I: Input extract. C: Control antibody. S: SFPQ/PSF specific antibody.

**Figure 3:**
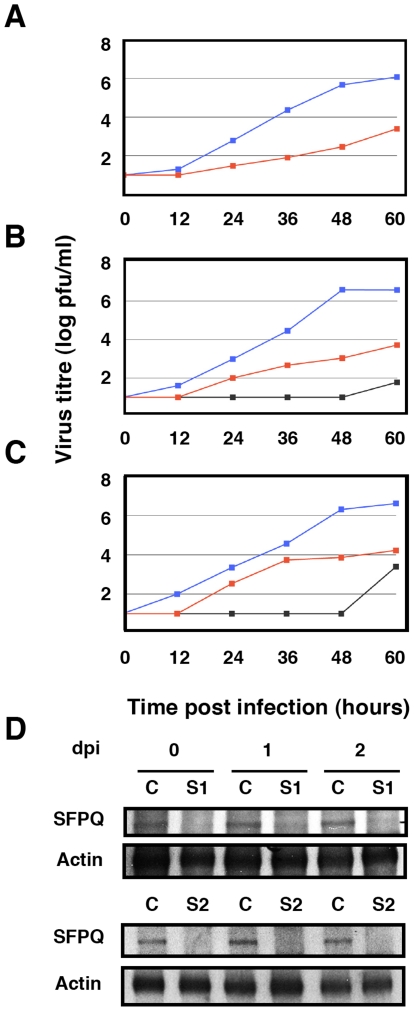
Kinetics of influenza virus multiplication in SFPQ/PSF-silenced cells. Cultures of human A549 cells were transfected with SFPQ/PSF-specific or control siRNAs as described under Materials and Methods and then infected with influenza virus at a moi of 0.001 pfu/cell. Aliquots of the supernatant media were withdrawn at the times indicated and used for virus titration by plaque-assay. The results presented are representative of two/three independent experiments. (A) Cultures were silenced with control siRNA (blue) or siRNA-1 (red) and infected with the VIC strain. (B) Cultures were silenced with control siRNA (blue), siRNA-1 (red) or siRNA-2 (grey) and infected with a recombinant virus containing the M, HA and NA segment of WSN in the background of VIC strain. (C) Cultures were silenced with control siRNA (blue), siRNA-1 (red) or siRNA-2 (grey) and infected with WSN strain. (D) At the times after infection indicated (days), the accumulation of SFPQ/PSF was determined in the cultures transfected with control (C) or SFPQ/PSF siRNAs (S1 or S2, as indicated) by Western-blot using anti SFPQ/PSF antibodies.

In previous studies we analysed the localisation of SFPQ/PSF and NP by confocal immunofluorescence of cells infected with VIC influenza virus. A clear co-localisation was observed at 4–6 hours post-infection (hpi), particularly prominent at the periphery of the cell nucleus [34]. To further analyse this association in infected cells we carried out co-immunoprecipitation experiments. Cultures of A549 cells were infected at high moi with the VIC strain of influenza virus and at various times after infection cell extracts were prepared and used for immunoprecipitation with anti-SFPQ/PSF or control antibodies. The immunoprecipitates were analysed by Western-blotting with antibodies specific for SFPQ/PSF and NP. The results are presented in Figure 2 and a clear co-immunoprecipitation of NP was observed with the SFPQ/PSF-specific antibodies. In view of these results, the relevance of SFPQ/PSF for influenza virus multiplication was then studied by gene silencing. Cultures of A549 cells were transfected with SFPQ/PSF-specific siRNA, or a scrambled unspecific siRNA as a control, and then infected with VIC influenza virus at low multiplicity. Samples were withdrawn from the supernatant medium at various times after infection and the virus titre was determined by plaque-assay on MDCK cells. The results of kinetics experiments run in triplicate are presented in Figure 3A. A protracted virus growth kinetics was apparent in the SFPQ/PSF–silenced cultures as compared to cultures transfected with control siRNA and the final titre was reduced by 2–3 log units in various experiments similar to that presented in Figure 3A. The level of SFPQ/PSF down-regulation was verified at various times after siRNA transfection and virus infection and is presented in Figure 3D. These results suggested that SFPQ/PSF plays an important role during influenza virus infection. To verify that the virus growth inhibition is really due to SFPQ/PSF down-regulation and not to an spurious off-target effect of the particular siRNA used, similar experiments were performed with an independent SFPQ/PSF-specific siRNA and the results are presented in Figure 3B and 3C. Furthermore, two additional virus strains were used in these experiments, WSN and a VIC/WSN recombinant virus. Again, a strong reduction in the yield of virus production was obtained, thereby confirming that SFPQ/PSF in an important human host factor for the multiplication of influenza virus.

**Figure 4:**
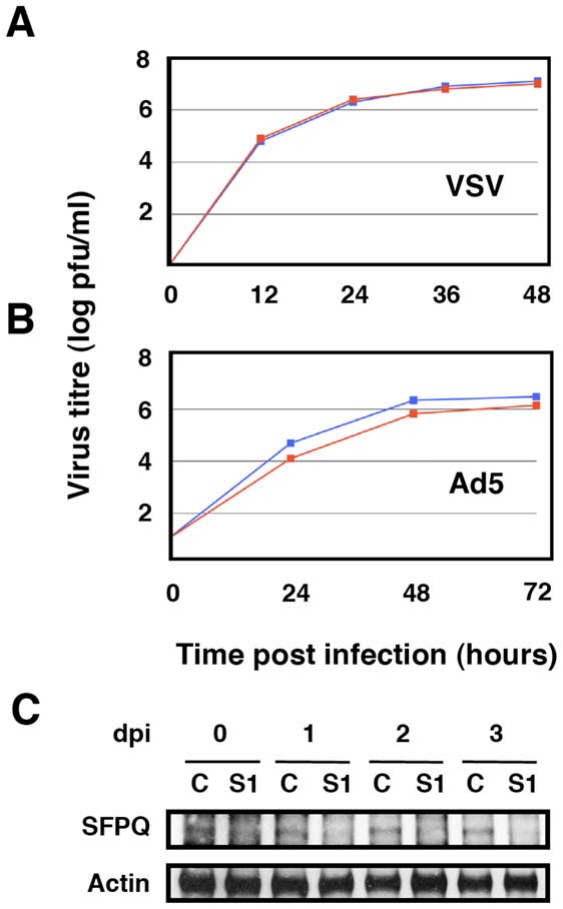
Kinetics of VSV and Ad5 multiplication in SFPQ/PSF-silenced cells. Cultures of human A549 cells were transfected with SFPQ/PSF-specific siRNA-1 (S1) or control (C) siRNAs, infected with VSV or Ad5 as described under Materials and Methods and virus titration was performed with samples obtained at the times indicated. The results presented are representative of two/three independent experiments. (A) Cultures were silenced with control siRNA (blue) or siRNA-1 (red) and infected with VSV at a moi of 0.001 pfu/cell. (B) Cultures were silenced with control siRNA (blue) or siRNA-1 (red) and infected with Ad5 at a moi of 2 pfu/cell. (C) At the times after infection indicated (days), the accumulation of SFPQ/PSF was determined in the cultures transfected with control (C) or SFPQ/PSF siRNAs (S1) by Western-blot using anti SFPQ/PSF antibodies.

As SFPQ/PSF is a multifunctional protein involved in many steps of cellular transcription and post-transcriptional RNA processing [37], it is conceivable that its down-regulation indirectly leads to reductions in influenza virus multiplication. For instance, it would be conceivable that SFPQ/PSF down-regulation inhibits cellular transcription and/or splicing to a level that makes influenza virus unable to replicate, as it depends on these processes for its own transcription and gene expression. Such a possibility would seem unlikely, as the general pattern of cellular protein synthesis is not altered by SFPQ/PSF silencing (see below), but we carried out controls to ascertain the specificity of SFPQ/PSF requirement for influenza virus multiplication. The multiplication of two additional viruses was studied in SFPQ/PSF-silenced cells: Vesicular stomatitis virus (VSV), as an additional example of negative-stranded RNA virus, and Adenovirus 5 (Ad5), as a nuclear virus that is strongly dependent on the cellular transcriptional and splicing machineries. Cultures of A549 cells were SFPQ/PSF- or control-silenced and infected with either VSV or Ad5 and the virus accumulated in the culture supernatant (VSV) or the infected cells (Ad5) was determined by plaque-assay on BHK21 (VSV) or HEK293T cells (Ad5). As presented in Figure 4A and 4B, the multiplication of neither virus was affected by the down-regulation of SFPQ/PSF, indicating that this human protein is a host factor specifically important for influenza virus multiplication.

**Figure 5:**
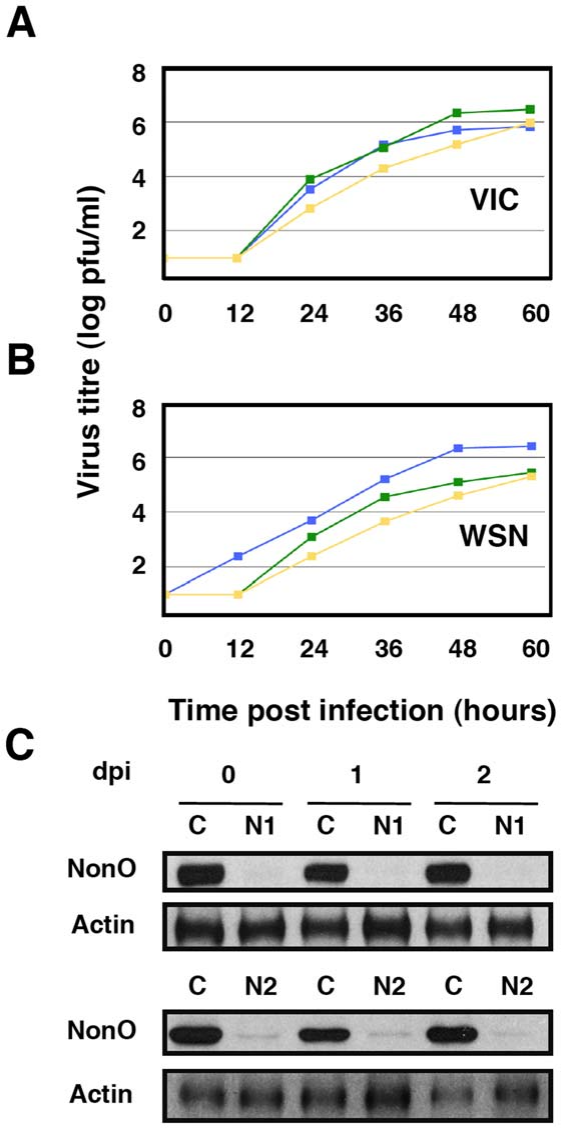
Kinetics of influenza virus multiplication in NonO-silenced cells. Cultures of human A549 cells were transfected with NonO-specific (N1 or N2, as indicated) or control (C) siRNAs as described under Materials and Methods and then infected with influenza virus at a moi of 0.001 pfu/cell. Aliquots of the supernatant media were withdrawn at the times indicated and used for virus titration by plaque-assay. The results presented are representative of two/three independent experiments. (A) Cultures were silenced with control siRNA (blue), siRNA N1 (green) or siRNA N2 (yellow) and infected with the VIC strain. (B) Cultures were silenced as above and infected with WSN virus. (C) At the times after infection indicated (days), the accumulation of NonO/p54 was determined in the cultures transfected with control (C) or SFPQ/PSF siRNAs (N1 or N2, as indicated) by Western-blot using anti NonO/p54 antibodies.

Since it has been shown that SFPQ/PSF associates to p54nrb/NonO (see above), it was important to ascertain whether influenza virus requires SFPQ/PSF or the SFPQ/PSF-p54nrb/NonO heterodimer for proper multiplication. Hence, we analysed the multiplication of influenza virus in A549 cells after silencing p54nrb/NonO by transfection of a p54nrb/NonO-specific siRNA. Importantly, silencing of p54nrb/NonO did not alter the accumulation of SFPQ/PSF or vice versa (data not shown). As indicated in Figure 5, no reduction in virus yield was observed when using the VIC virus strain (Figure 5A) and a small reduction in WSN virus amplification was observed by down-regulation of p54nrb/NonO, much more limited than that observed when silencing SFPQ/PSF (Figure 5B), in spite of an almost complete block of p54nrb/NonO expression (Figure 5C). Therefore, we conclude that it is SFPQ/PSF by itself what influenza virus requires carrying out a normal infection cycle.

### Down-regulation of SFPQ/PSF reduces virus RNA replication and gene expression

**Figure 6:**
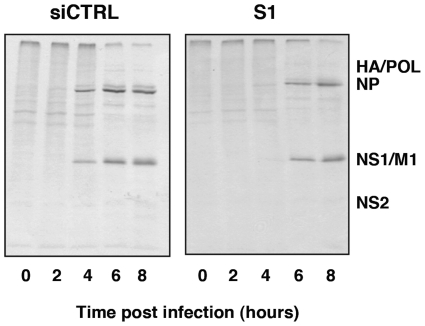
Kinetics of influenza virus protein synthesis in SFPQ/PSF-silenced cells. Cultures of human A549 cells were transfected with SFPQ/PSF-specific siRNA-1 (S1) or control siRNA (siCTRL) as described under Materials and Methods and then infected with VIC virus at a moi of 3 pfu/cell. At the times in hours indicated the cultures were pulse-labelled with ^35^S-met-cys and total protein extracts were prepared. The samples were analysed by polyacrylamide gel electrophoresis and autoradiography. The mobility of some of the virus-specific proteins is indicated to the right.

Once the relevance of SFPQ/PSF for influenza virus infection was verified, we analysed the role of this host factor in the virus cycle. First, the synthesis of viral proteins was studied in SFPQ/PSF- and control-silenced A549 cells. Cultures of SFPQ/PSF- or control-silenced A549 cells were infected at high multiplicity and pulse-labelled with ^35^S-met-cys at various times after infection. The labelled proteins were analysed by polyacrylamide gel electrophoresis and autoradiography and the results are presented in Figure 6. The synthesis of the major virus proteins was reduced and delayed in SFPQ/PSF-silenced cells, indicating that SFPQ/PSF down-regulation leads to a general reduction and delay in virus gene expression. However, it is worth mentioning that only a slight change was observed in the level and pattern of cellular protein synthesis upon SFPQ/PSF silencing (compare lanes 0 in siCRTL and S1 panels in Figure 6). Similar results were obtained when the accumulation of viral proteins was determined by Western-blot (Figure S1).

**Figure 7:**
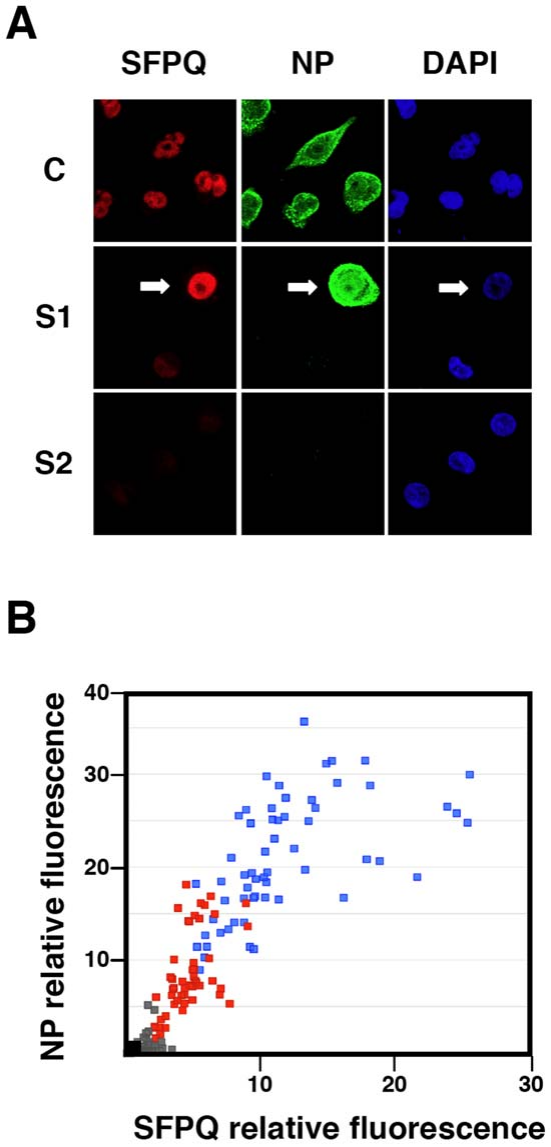
Cytological analysis of influenza virus infection of SFPQ/PSF-silenced cells. Cultures of human A549 cells were transfected with control siRNA (C) or SFPQ/PSF-specific siRNAs (S1 or S2 as indicated), as described under Materials and Methods, and then infected with VIC virus at a moi of 3 pfu/cell. The cultures were fixed at 6 hpi and processed for immunofluorescence using antibodies specific for SFPQ/PSF and NP. (A) The images presented are representative confocal sections showing the presence of nuclei (DAPI –blue-), SFPQ/PSF (red) and NP (green). (B) Graphical representation of the NP (vertical axis) and SFPQ/PSF (horizontal axis) signals obtained for random 1024x1024 images of each sample. Blue: Control-silenced cultures. Red: SFPQ/PSF-silenced cultures (S1). Grey: SFPQ/PSF-silenced cultures (S2). The black dot indicates the background signals obtained for cultures stained with the secondary antibodies only.

To analyse further the phenotype of the infection cycle in SFPQ/PSF-silenced cells, the localisation of progeny RNPs was studied by confocal immunofluorescence with anti-NP antibodies. The results are presented in Figure 7. As expected, the level of SFPQ/PSF was decreased in SFPQ/PSF-silenced cells and a general reduction in NP signal was observed, as compared with infected, control-silenced cells (Figure 7A). Only cells with a level of SFPQ/PSF similar to that of control-silenced cells showed an accumulation of NP comparable to control infected cells, although the localisation was not normal (Figure 7A; arrow). Significantly, a linear correlation was observed between the immunofluorescence signals of SFPQ/PSF and NP in random fields of control- or SFPQ/PSF-silenced cells (Figure 7B). Thus, the low levels of virus protein synthesis (Figure 6) and the small virus production (Figure 3) observed in SFPQ/PSF-silenced cultures could be simply the consequence of the infection of a small number of cells not completely silenced upon transfection of SFPQ/PSF-specific siRNA.

### Viral transcription, but not splicing, is affected in SFPQ/PSF silenced cells

**Figure 8:**
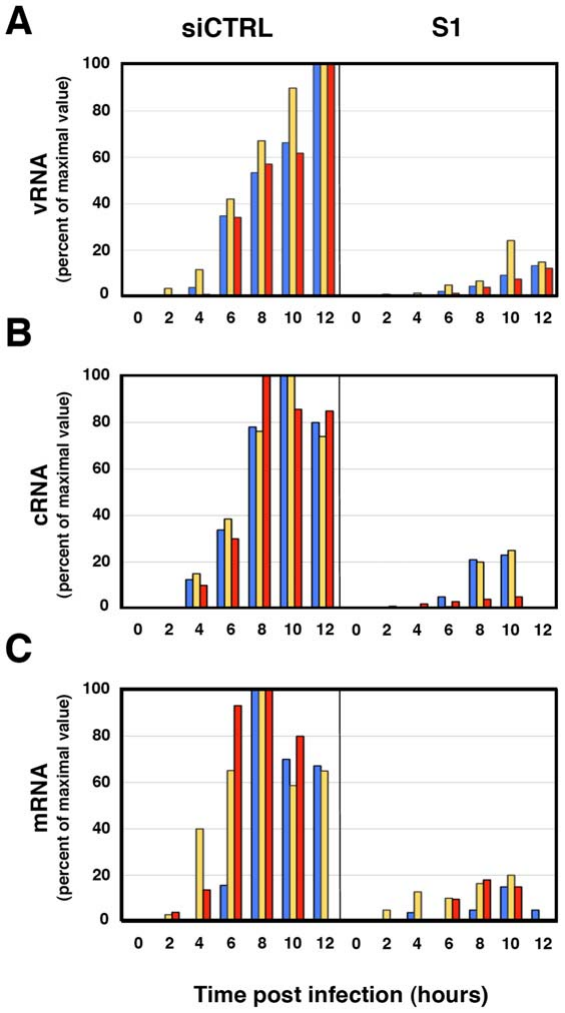
Accumulation of positive- and negative-polarity RNAs during the influenza virus infection of SFPQ/PSF-silenced cells. Cultures of human A549 cells were transfected with SFPQ/PSF-specific (S1) or control siRNAs (siCTRL) as described under Materials and Methods and then infected with influenza virus at a moi of 3 pfu/cell. Total cell RNA was isolated at the times indicated and used for determination of vRNA (A) cRNA (B) or mRNA (C) by hybridisation with probes specific for NP (blue), NS (yellow) and M (red). The data is presented as percent of maximal values and is representative of three independent kinetics experiments.

**Figure 9:**
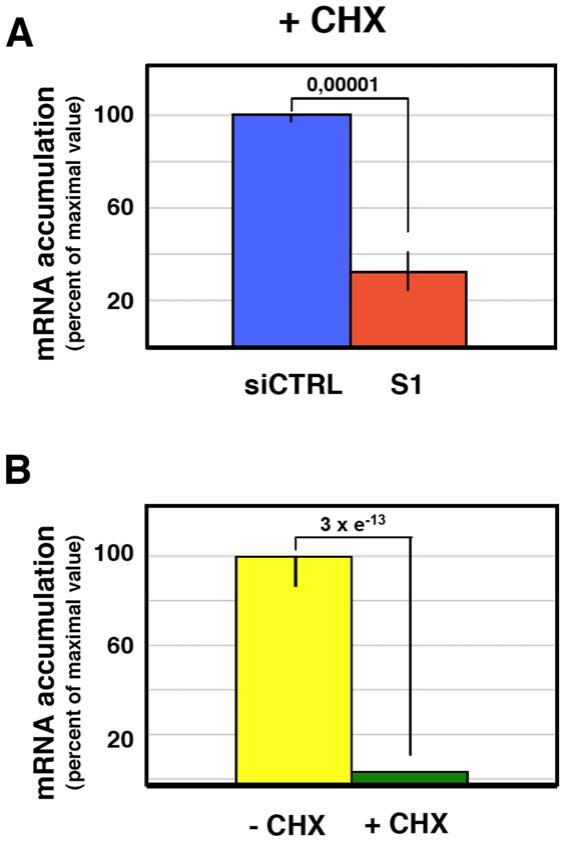
Accumulation of mRNA during the influenza virus infection of SFPQ/PSF-silenced cells treated with cycloheximide. Cultures of human A549 cells were transfected with SFPQ/PSF-specific (S1) or control siRNAs (siCTRL) as described under Materials and Methods and then infected with influenza virus at a moi of 3 pfu/cell for 6 hours in the presence or absence of cycloheximide (100 µg/ml). The accumulation of viral RNA was determined by RT-qPCR using probes specific for the NS segment. Significance was determined by the Student's t test. (A) The accumulation of viral RNA in control and SFPQ/PSF-silenced cells treated with cycloheximide is presented as average and standard deviation of 6 determinations. (B) The accumulation of mRNA in infected cells treated or not with cycloheximide is presented as average and standard deviation of 6 determinations.

The reduction of virus protein synthesis under conditions of SFPQ/PSF down-regulation might be due to defects in genome amplification, virus transcription, splicing or translation of viral mRNA. As SFPQ/PSF has been described as a splicing factor, we first analysed whether its down-regulation would alter the splicing of virus mRNAs. The amounts of NS1 and NS2 mRNAs were determined in control and SFPQ/PSF silenced infected cells by a RT-qPCR procedure using TaqMan probes (Table S1). The proportion of NS1 versus total NS mRNA was indistinguishable in both experimental conditions (ratio control/silenced cells 1.016+/−0.035; n = 7 experiments). Next, the levels of accumulation of vRNA, cRNA and mRNA were determined in SFPQ/PSF-silenced and control-silenced cells. Total cell RNA was isolated at various times after high-multiplicity infections and the amounts of virus-specific RNAs corresponding to the NP, NS and M virus segments were determined by hybridisation with specific probes. The results of a representative experiment are presented in Figure 8. In control-silenced cells, the kinetics of accumulation of virus RNAs showed a pattern analogous to that previously described [56], [57], while in SFPQ/PSF-silenced cells a protracted kinetics was observed and 4-5 fold reductions in maximal accumulations of vRNA, cRNA and mRNA were determined. These results indicated that SFPQ/PSF is required for virus RNA replication but could not tell whether this was a direct effect and whether SFPQ/PSF also played a role in virus transcription. To clarify these questions, the accumulations of primary virus transcripts were determined after infection of SFPQ/PSF-silenced and control-silenced cells. The cells were infected at high multiplicity in the presence of cycloheximide to avoid the synthesis of viral proteins and hence the replication of viral RNA [58]. The accumulation of total NS transcripts was determined by RT-qPCR and demonstrated that silencing SFPQ/PSF leads to a 3-fold reduction in primary transcription (Figure 9A). To verify the inhibition of virus multiplication, virus mRNA was determined in infected cells treated or not with cycloheximide. The results are presented in Figure 9B and document around 50-fold reduction upon treatment with the drug.

**Figure 10:**
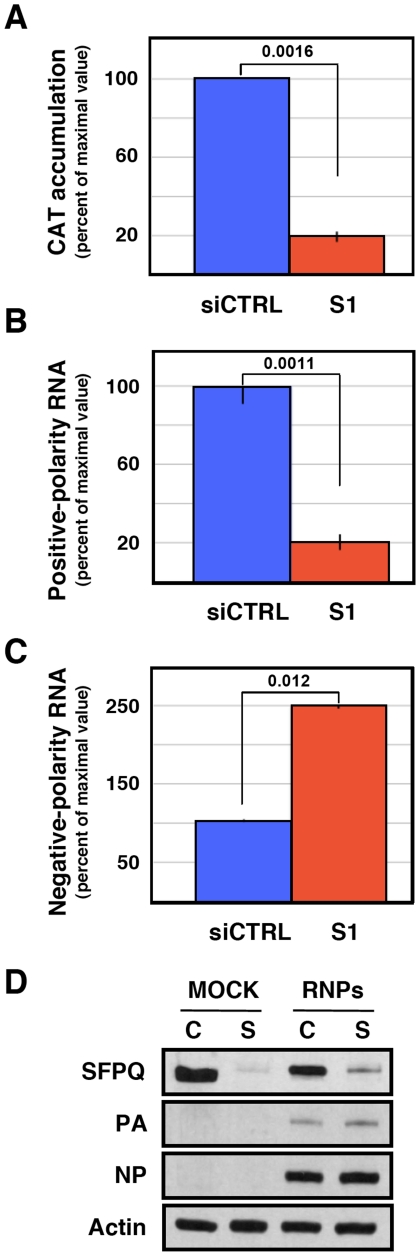
Accumulation of positive- and negative-polarity RNA in a recombinant replicon system. Cultures of HEK293T cells were transfected with SFPQ/PSF-specific (S1) or control siRNAs (siCTRL) as described under Materials and Methods. When the expression of SFPQ/PSF was known to be down-regulated, the cultures were further transfected with plasmids expressing the polymerase subunits and the NP, as well as with a genomic plasmid expressing a pseudoviral gene containing the cat gene in negative polarity. As control, the cultures were transfected with empty plasmids. At 24 hours post plasmid transfection total cell extracts were prepared and total cell RNA was isolated. Significance was determined by the Student's t test. (A) The accumulation of CAT protein is indicated as percent of maximal values. (B) The accumulation of positive-polarity RNA was determined by hybridisation with CAT-specific probes and is presented as percent of maximal values. (C) The accumulation of negative-polarity RNA was determined by hybridisation with CAT-specific probes and is presented as percent of maximal values. The values presented represent the average and standard deviation of 3 independent transfection experiments. (D) The silencing of SFPQ was controlled by Western-blot, using actin as loading control. The level of expression of PA and NP was ascertained by Western-blot.

These results suggest that SFPQ/PSF might simply be required for virus primary transcription and the observed reduction in vRNA accumulation would be an indirect consequence, since viral protein expression is essential for viral RNA replication [58]. To test whether virus RNA replication is directly inhibited by SFPQ/PSF down-regulation, in addition to the observed inhibition of primary transcription, we made use of a recombinant replicon system to analyse RNA replication and secondary transcription. In this system, no primary transcription is required for RNA replication to take place, as the virus proteins are provided by plasmid expression in *trans*. Human HEK293T cells were transfected with SFPQ-specific or control siRNAs and later transfected with plasmids encoding the virus polymerase subunits, NP and a virus genomic plasmid encoding the *cat* gene in negative polarity. The down-regulation of SFPQ/PSF was ascertained by Western-blot (Figure 10D) and the CAT protein accumulation was determined as a reporter of total replicon activity. The results indicated that silencing SFPQ/PSF lead to a 5-fold reduction in CAT expression (Figure 10A). To determine whether this reduction was due to alterations in viral RNA replication, transcription or both, total cell RNA was isolated and used to determine negative-polarity and positive-polarity RNA accumulations by hybridisation with specific probes. As shown in Figure 10B, a 5-fold reduction in viral transcription was apparent, whereas a two-fold increase was observed between the accumulations of vRNA in control or SFPQ/PSF-silenced cells (Figure 10C). These results indicated that SFPQ/PSF is required for viral transcription, but not for virus RNA replication and suggest that, in the absence of SFPQ/PSF, the viral RNPs are preferentially devoted to RNA replication instead of transcribing their template.

### Depletion of SFPQ/PSF leads to diminished production of viral polyadenylated RNA *in vitro*

**Figure 11:**
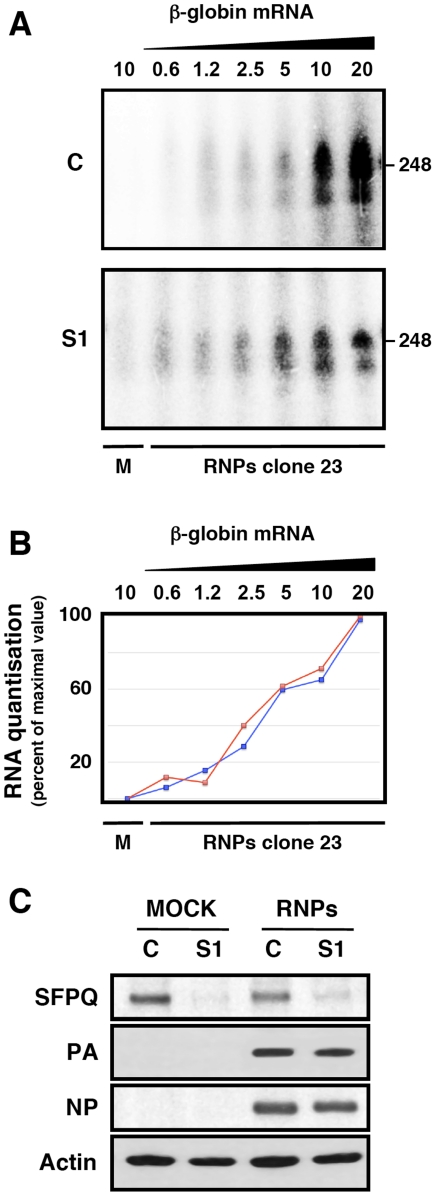
Cap-donor dose-response for *in vitro* transcription of recombinant RNPs. Cultures of HEK293T cells were transfected with SFPQ/PSF-specific (S1) or control siRNAs (siCTRL) as described under Materials and Methods. When the expression of SFPQ/PSF was known to be down-regulated, the cultures were further transfected with plasmids expressing the polymerase subunits and the NP, as well as with a genomic plasmid expressing a deleted NS RNA segment (clone 23) [2] in negative polarity. Extracts of these cultures were used for *in vitro* transcription using increasing amounts of ß-globin mRNA as a cap donor and the transcripts were analysed by denaturing polyacrylamide gel electrophoresis. (A) Denaturing polyacrylamide gel electrophoresis of in vitro transcripts. M denotes a mock-reconstitution of recombinant RNPs in which pcDNA3 plasmid was transfected. The concentrations (µg/ml) of ß-globin mRNA used as cap-donor are indicated to the top. The mobility of a genome-size marker of 248 nt is indicated to the right. (B) The quantisation of the results presented in A is presented as percent of maximal value in each series. Blue line: Control-silenced extract. Red line: SFPQ/PSF-silenced extract. (C) The down-regulation of SFPQ/PSF by silencing was verified by Western-blot with specific antibodies. The level of expression of PA and NP was ascertained by Western-blot.

**Figure 12:**
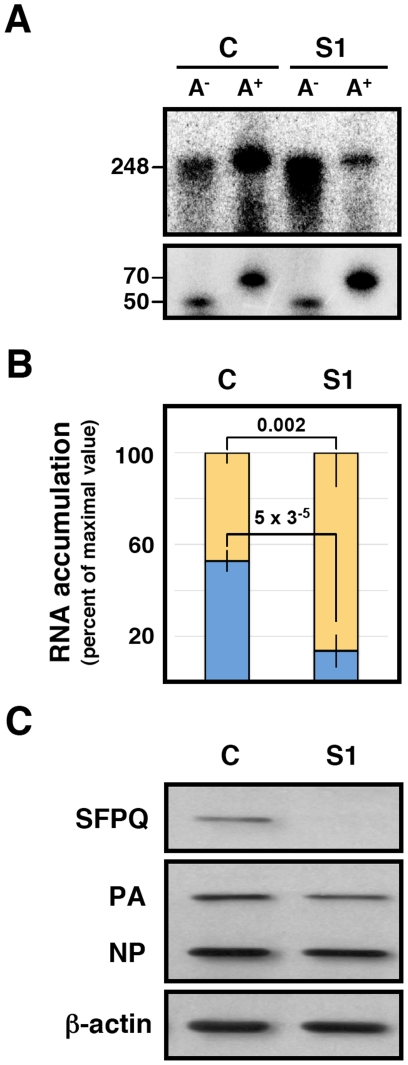
Dependence of SFPQ/PSF for the *in vitro* polyadenylation of transcripts. Recombinant RNPs were generated in control-silenced (C) or SFPQ/PSF-silenced (S1) HEK293T cells and in vitro transcription was performed as indicated in the legend to Figure 11. The transcripts were separated into poly A^+^ and poly A^−^ fractions, using a non-polyadenylated oligonucleotide (50 nt) and a polyadenylated oligonucleotide (70 nt) as recovery probes, and the fractionated transcripts were analysed by electrophoresis on denaturing polyacrylamide gels. (A) Representative results of five independent experiments. The positions of a 248 nt marker identical to the clone 23 genome, as well as the recovery probes are indicated to the left. (B) Quantification of the proportion of the poly A^+^ (blue) and poly A^−^ (yellow) transcripts after normalisation for recovery. The data represent averages and standard deviations of five experiments. Significance was determined by the Student's t test. (C) The silencing of SFPQ was controlled by Western-blot, using actin as loading control. The level of expression of PA and NP was ascertained by Western-blot.

One possible mechanism to explain the effects of SFPQ/PSF down-regulation on influenza virus transcription would imply that the interaction of SFPQ/PSF with the viral polymerase present in the RNP increases the affinity of the enzyme for the cap structure. The apparent Kd for the interaction of the isolated PB2 cap-binding domain with 7mGTP is around 170 µM [10], in good agreement with the inhibition data reported for cap-RNA crosslinking to influenza virus RNPs [59], whereas the affinity of binding of eIF4E or CBC to cap-analogues is much higher [60], [61]. This is in contradistinction to the elution profiles of eIF4F and influenza polymerase-template complexes on a 7mGTP-Sepharose resin [14] which show a stronger binding of the latter. Hence, it is conceivable that some cellular factor(s), for instance SFPQ/PSF, interact with viral polymerase and enhances its affinity for binding to cap. To test such possibility we generated recombinant RNPs by transfection of HEK293T cells, which had previously been either control- or SFPQ/PSF-silenced. Total cell extracts of these cells were used for *in vitro* transcription using ß-globin mRNA as cap-donor. A wide concentration range of cap-donor was employed to test for a potential difference in cap-donor dose response when the reaction was performed in the presence or absence of SFPQ/PSF protein. The results are presented in Figure 11 and no change in the profile of virus RNA synthesis was apparent when SFPQ/PSF was silenced (Figure 11B). However, a clear change in the size distribution of RNA products was observed depending on the downregulation of SFPQ/PSF and irrespective of the cap-donor concentration (Figure 11A). In the presence of SFPQ/PSF the RNA profile was reminiscent of a polyadenylated virus mRNA, as the transcript size was slightly larger than the template (used in the gel as a marker) while downregulation of SFPQ/PSF led to a variety of RNA sizes, always smaller than the template. To further characterise the transcripts generated with SFPQ/PSF-silenced samples they were separated into poly A^+^ and poly A^−^ fractions and analysed by denaturing polyacrylamide gel electrophoresis. The results are presented in Figure 12. A consistent reduction in the amount of polyadenylated RNA was observed when SFPQ/PSF was downregulated, with a corresponding increased in the poly A^−^ fraction (Figure 12A). Quantification of 5 experiments indicated that the total amount of transcript was not affected by SFPQ/PSF downregulation but the fraction of poly A^+^ viral mRNA was reduced about 4–5 fold (Figure 12 B). Similar results were obtained when other SFPQ/PSF-specific siRNA was used (see Figure S2). A considerable fraction of the viral poly A^−^ transcrips showed sizes smaller than the template, consistent with RNA degradation or premature termination but, interestingly, the profile of these poly A^−^ transcripts was identical for control- or SFPQ/PSF-silenced samples, suggesting that the reduction in poly A^+^ transcripts is not due to a defect in transcript elongation but most probably to a deficiency in the polyadenylation step.

### Concluding remarks

The results presented here show that SFPQ/PSF is specifically required for influenza virus multiplication and indicate that this cellular factor is essential for the transcription of viral RNPs during both primary and secondary mRNA synthesis. Furthermore, the results shown suggest that SFPQ/PSF plays a role during the polyadenylation step in virus transcription. With the evidence presented, the following picture could be envisaged upon SFPQ down-regulation in infected cells: The primary viral mRNAs lacking poly A would be unstable and hence their accumulation diminished as compared to infections performed in normal cells. In addition, normal recycling of the transcribing polymerase could be affected. As a consequence of these effects, viral RNA replication would be indirectly inhibited, since little polymerase and NP would be synthesised. Moreover, a similar inhibition could be predicted for secondary transcription of the small amount of viral progeny RNA, with the final result of very low viral gene expression and virus production.

At present it is not clear how SFPQ/PSF participates in the polyadenylation step of viral transcription. The available evidence indicates that polyadenylation of viral mRNAs is carried out by the polymerase by reiterative copy of the oligo U signal located close to the 5′-terminus of the vRNA template [62]–[65] and is mechanistically distinct from the cellular cleavage and polyadenylation process. The SFPQ/PSF protein is an RNA-binding protein that has been described as essential for the formation of the spliceosome and can be cross-linked to the pre-mRNA in the spliceosome [46]. Furthermore, purified SFPQ/PSF can be specifically cross-linked to poly U, but not to poly C, A or G, showing the same sequence specificity than PTB [38]. Therefore, it is tempting to speculate that SFPQ/PSF could interact both with the viral polymerase [34] and with the viral polyadenylation signal within the RNP to promote polymerase stuttering at the site. Further experiments will be required to test this and other possible alternatives.

Down-regulation of SFPQ/PSF leads to a dramatic decrease in the yield of virus infection and hence it could be considered as a new target for antiviral design, a particularly interesting one as it is involved in a very early stage of the infection. Silencing of mouse SFPQ/PSF leads to chromosome instability [66] and we have verified that down-regulation of SFPQ/PSF in some human cells strongly reduces their growth kinetics (unpublished results). Therefore, one should aim at blocking the association of SFPQ/PSF with virus polymerase/RNP for the design of potential new antivirals.

## Materials and Methods

### Biological materials

The HEK 293T cell line [67] was obtained from J.C. de la Torre and the A549 human cell line [68] was obtained from J.A. Melero. The MDCK and BHK21 cell lines were purchased from ATCC. Cell culture was carried out as described [69]. The influenza virus strains A/Victoria/3/75 (H3N2) (VIC), WSN (H1N1) and a recombinant of both strains (VIC/WSN) was used in these experiments. Titrations were done in MDCK cells as described [70]. Vesicular stomatitis virus (VSV) was provided by R. Alfonso and titrated in BHK21 cells. Adenovirus 5 was provided by P. Fortes. Virus stocks were prepared and titrated in HEK 293T cells as described [71].

Plasmids pCMVPB1, pCMVPB2, pCMVPA and pCMVNP, expressing the influenza virus polymerase subunits and the NP have been described [57]. Plasmid pHHCAT, that transcribes a virus-like *cat* gene in negative polarity, was provided by A. Rodríguez. The monoclonal antibodies specific for PA have been described [72], [73]. A monoclonal antibody specific for the N-terminal region of M1 and M2 proteins [74] was provided by A. García-Sastre. Antisera specific for PB1 and NP were generated by immunisation of rabbits with recombinant proteins [2], [14], [31]. A monoclonal antibody specific for SFPQ/PSF (ab11825) and polyclonal sera specific for GAPDH (ab9485) and actin (ab8226) were purchased to Abcam. The secondary antibodies used for Western-blot and immunofluorescence was purchased to Sigma and Invitrogen, respectively. Total mouse IgG, with no known specificity, was used as immunoprecipitation control.

### Protein analyses

Protein samples were separated by polyacrylamide-SDS gel electrophoresis and transferred to Immobilon filters. Western-blotting was carried out essentially as described [75]: The membranes were saturated with 3% BSA for 1 h and then incubated with the primary antibodies for 1 h at room temperature. The filters were washed with PBS containing 0.25% Tween 20 and incubated with the appropriate secondary antibody conjugated to horseradish peroxidase. After further washing as above the filters were developed by enhanced chemiluminiscence. For immunoprecipitations, extracts from mock-infected or infected cells were incubated with goat-antimouse-agarose beads (A6531, Sigma) loaded with a monoclonal antibody specific for SFPQ/PSF or an equal amount of total mouse IgG as negative control. After extensive washing with a buffer containing 50 mM Tris HCl pH 7,5, 150 mM NaCl, 0,5% NP-40, 1,5 mM MgCl2, 1 mM DTT, 1 u/µl HPRI and protease inhibitors containing EDTA, the beads were extracted by boiling with Laemmli loading buffer and the samples were analysed by Western-blot with antibodies specific for SFPQ/PSF and NP. For immunofluorescence, cells were fixed with 3% paraformaldehyde. The cultures were permeabilised with 0.5% Triton X100 and processed for indirect immunofluorescence as described before [31]. Images were collected on a Leica SP5 confocal microscope (Leica Microsystems) and processed with the LAS AF Software (Leica Microsystems). For quantisation of cellular staining with anti-SFPQ/PSF and NP antibodies, the average intensities of 50 random images (1024×1024 pixels) of each preparation were determined using the LAS AF Software. The procedures for protein labelling *in vivo* have been described [76]. Cultures were washed, incubated for 1 h in a DMEM medium lacking met-cys and labelled with ^35^S-met-cys to a final concentration of 200 µCi/ml. After incubation for 1 h, total extracts were prepared in Laemmli sample buffer and processed by polyacrylamide gel electrophoresis and autoradiography. Quantisation of CAT protein in total cell extracts was done by ELISA (GE Healthcare).

### RNA analyses

The amplification *in vivo* of recombinant RNPs was performed essentially as described [77]. In brief, cultures of HEK293T cells (2.5 10^6^ cells) were transfected with a mixture of plasmids expressing the polymerase subunits (pCMVPB1, 12.5 ng; pCMVPB2, 12.5 ng; pCMVPA, 2.5 ng), NP (pCMVNP, 500 ng) and a genomic plasmid expressing a vRNA-like *cat* gene (pHHCAT, 500 ng), using the calcium phosphate technique [78]. At 24 hours post-transfection, total cell extracts were prepared for CAT determination or total cell RNA was extracted. For RNA extraction cell pellets were resuspended in 1 ml of TRIZOL reagent (Invitrogen) and the RNA was purified as recommended by the manufacturer. The RNA was digested with RNAse-free DNAse (1 u/µg) for 1 h at 37°C, extracted with phenol-chloroform-isoamylalcohol and precipitated with ethanol. For alternative splicing studies, poly A^+^ RNA was isolated by two rounds of chromatography on oligo-dT-cellulose as described previously [79]. The purified RNA was resuspended in nuclease-free water and the absorbance was measured at 260 nm (NanoDrop ND-1000).

For dot-blot hybridisation, aliquots of purified RNAs were denatured for 15 min at 55°C in 10SSC, 7.5% formaldehyde and fixed on nylon filters by UV cross-linking. As controls, total yeast RNA or various amounts of plasmids containing cDNAs of the full-length virus genomic segments or the corresponding viral mRNAs, were fixed on the hybridisation filters. Hybridisation was carried out overnight at 40°C in 6xSSC, 0.5% SDS, 5× Denhardt's mixture 26–47% formamide, depending on the probe, and 100 µg/ml single-stranded DNA. After washing with 0.5xSSC-0.5% SDS at 40°C, the filters were quantified in a phosphorimager. As probes, synthetic oligonucleotides specific to detect the various RNA species of each RNA segment analysed were used (Table S1). They were labelled with gamma-^32^P-ATP and polynucleotide kinase. Additionally, specific riboprobes were used to specifically detect cRNAs. They were transcribed by T3 RNA polymerase using as templates synthetic DNAs containing T3 promoters fused to the viral sequences (Table S1).

For siRNA transfection, cultured A549 cells were incubated independently with 5 nM of siSFPQ 1 (107613), siSFPQ 2 (15923), specific for SFPQ, or siNonO 1 (s9614), siNonO 2 (s9612), specific for NonO, or an irrelevant siRNA (AM4611) from Ambion, using Lipofectamine (Invitrogen) as recommended by the manufacturer. Transfection was carried out twice on consecutive days to increase the silencing efficiency before infection.

Quantification of virus-specific RNAs for splicing and primary transcription analyses was carried out by RT-qPCR as follows: The RT reaction was performed by addition of 100 ng of RNA resuspended in 10 µl of nuclease-free water and 10 µl of Reaction Mix 2x (Applied Biosystems) as recommended by the manufacturer. From each 20-µl reaction, 2 µl of cDNA was transferred directly to 96-well PCR plates and 12,5 µl of TaqMan universal master mixture (Applied Biosystems) and 1,25 µl of Custom TaqMan assay (designed by Applied BioSystems) were added. PCR was carried out in a PRISM 7000 Sequence detection system (Applied Biosystems), with 1 cycle of 50°C for 2 min followed by 1 cycle of 95°C for 10 min, 40 cycles of 95°C for 15 s and 60°C for 1 min. The cycle threshold (Ct) was determined with analytical software (SDS; Applied Biosystems). Serial dilutions of cDNA were used to ensure amplification in the lineal range. To construct calibration curves for quantification, we generated PCR products whose sequences were identical to the spliced or unspliced mRNAs of NS segment. The sequences of TaqMan probes and primers are presented in Table S1. For *in vitro* transcription, extracts from control- or SFPQ/PSF-silenced HEK293T cells in which a mini-RNP was reconstituted [2] were incubated for 60 min at 30°C in 20 µl reactions containing 50 mM Tris.HCl, 100 mM KCl, 2 mM MgCl_2_, 1 mM DTT, 1 mM each of ATP, CTP and UTP, 1 µM alpha-^32^P-GTP (0.5 mCi/µmol), 10 µg/ml actinomycin D, 1 u/µl HPRI, pH 8.0 and µg/ml (or various amounts, depending on the experiment) ß-globin mRNA. The reaction products were phenol-extracted, ethanol-precipitated and analysed by electrophoresis on 6% polyacrylamide-7 M urea gels. The purified in vitro transcripts were separated into poly A^+^ and poly A^−^ fractions by chromatography on oligo dT-cellulose as described [80]. To monitor the recovery of RNAs during extraction and fractionation two labelled synthetic oligonucleotides were added, a 50 nt oligonucleotide lacking poly T sequences and a 70 nt long containing a 39T tract at its 3′-terminus.

## Supporting Information

Figure S1 **Accumulation of viral proteins during influenza virus infection of SFPQ/PSF-silenced cells.** Cultures of human A549 cells were transfected with SFPQ/PSF-specific (S1) or control siRNAs (siCTRL) as described under Materials and Methods and then infected with influenza virus at a moi of 3 pfu/cell. At the times after infection indicated, total cell extracts were prepared and used for Western-blot with the antibodies indicated to the right.
(TIF)

Figure S2 **Dependence of SFPQ/PSF for the** ***in vitro*** **polyadenylation of transcripts.** Recombinant RNPs were generated in control-silenced (C) or SFPQ/PSF-silenced (S2) HEK293T cells and in vitro transcription was performed as indicated in the legend to Figures 11 and 12. The transcripts were separated into poly A^+^ (blue) and poly A^−^ (yellow) fractions, using a non-polyadenylated oligonucleotide (50 nt) and a polyadenylated oligonucleotide (70 nt) as recovery probes, and the fractionated transcripts were analysed by electrophoresis on denaturing polyacrylamide gels. (A) Representative results of two independent experiments. The positions of a 248 nt marker identical to the clone 23 genome, as well as the recovery probes are indicated to the left. (B) Quantification of the proportion of the poly A^+^ and poly A^−^ transcripts after normalisation for recovery. The data represent averages and ranges of two experiments. (C) The silencing of SFPQ was controlled by Western-blot, using actin as loading control. The proper reconstitution of recombinant RNPs was ascertained by Western-blot with antibodies specific for PA and NP.
(TIF)

Table S1 **Oligonucleotides used as probes for detection of viral RNAs.** The Table shows the sequences of the oligonucleotides used as probes for hybridisation to vRNAs, mRNAs and cRNAs, corresponding to the M, NP and NS segments as indicated. The length of the probes and the concentration of formamide used during the hybridisation reactions are also indicated. Likewise, the sequences used for amplification of NS1 mRNA (NS1 intron), NS2 mRNA (NS2 border) and total NS mRNAs (Total NS) are indicated, as well as the corresponding Taqman probes.
(PDF)
