# Supplementary figures and images for: The Splicing Factor Proline-Glutamine Rich (SFPQ/PSF) Is Involved in Influenza Virus Transcription
Source: PLoS Pathog. 2011 Nov 17;7(11):e1002397. doi: 10.1371/journal.ppat.1002397 (PMC3219729; doi:10.1371/journal.ppat.1002397)

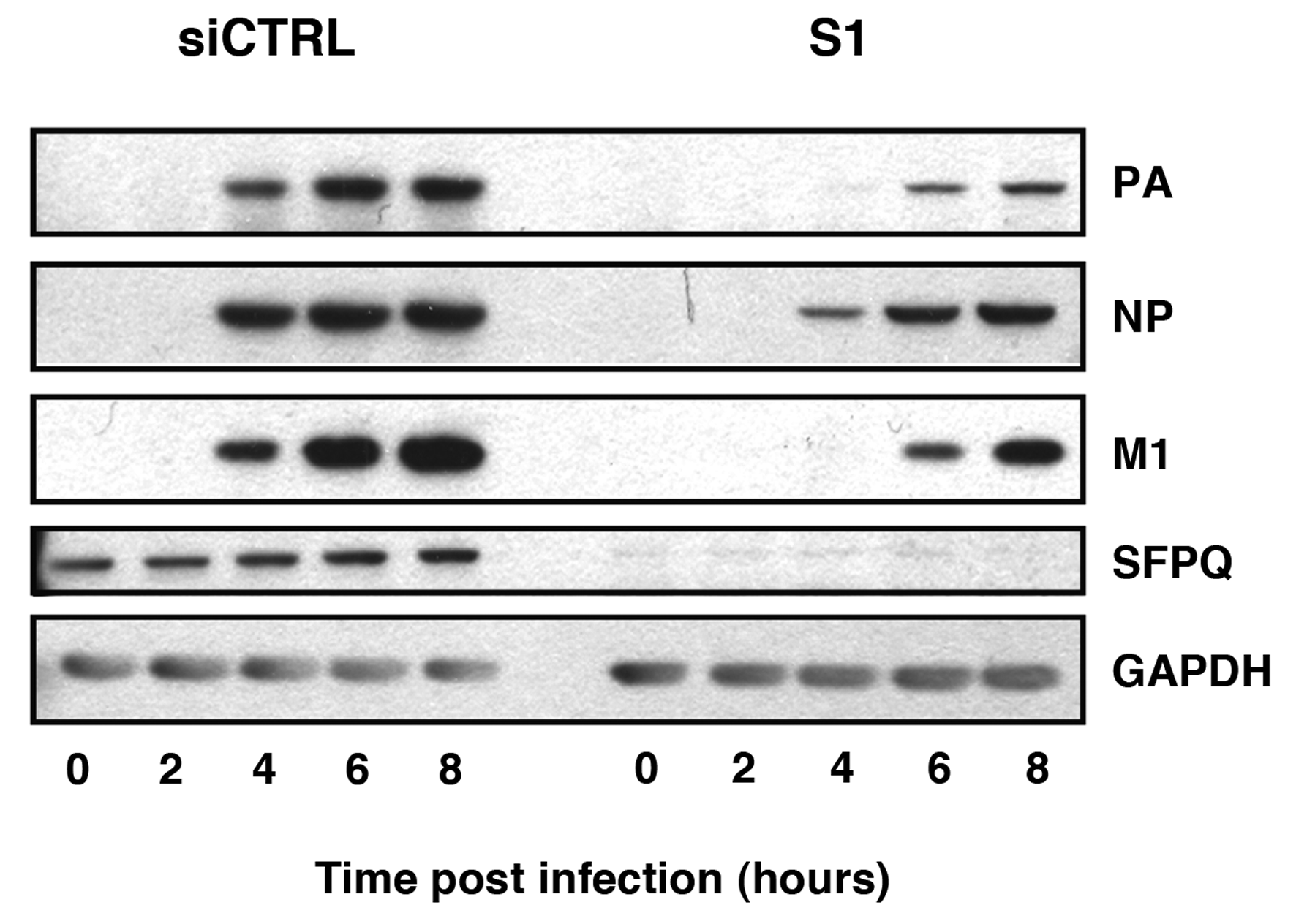

Supplement: Figure S1 — Accumulation of viral proteins during influenza virus infection of SFPQ/PSF-silenced cells. Cultures of human A549 cells were transfected with SFPQ/PSF-specific (S1) or control siRNAs (siCTRL) as described under Materials and Methods and then infected with influenza virus at a moi of 3 pfu/cell. At the times after infection indicated, total cell extracts were prepared and used for Western-blot with the antibodies indicated to the right. (TIF) [file ppat.1002397.s001.tif]

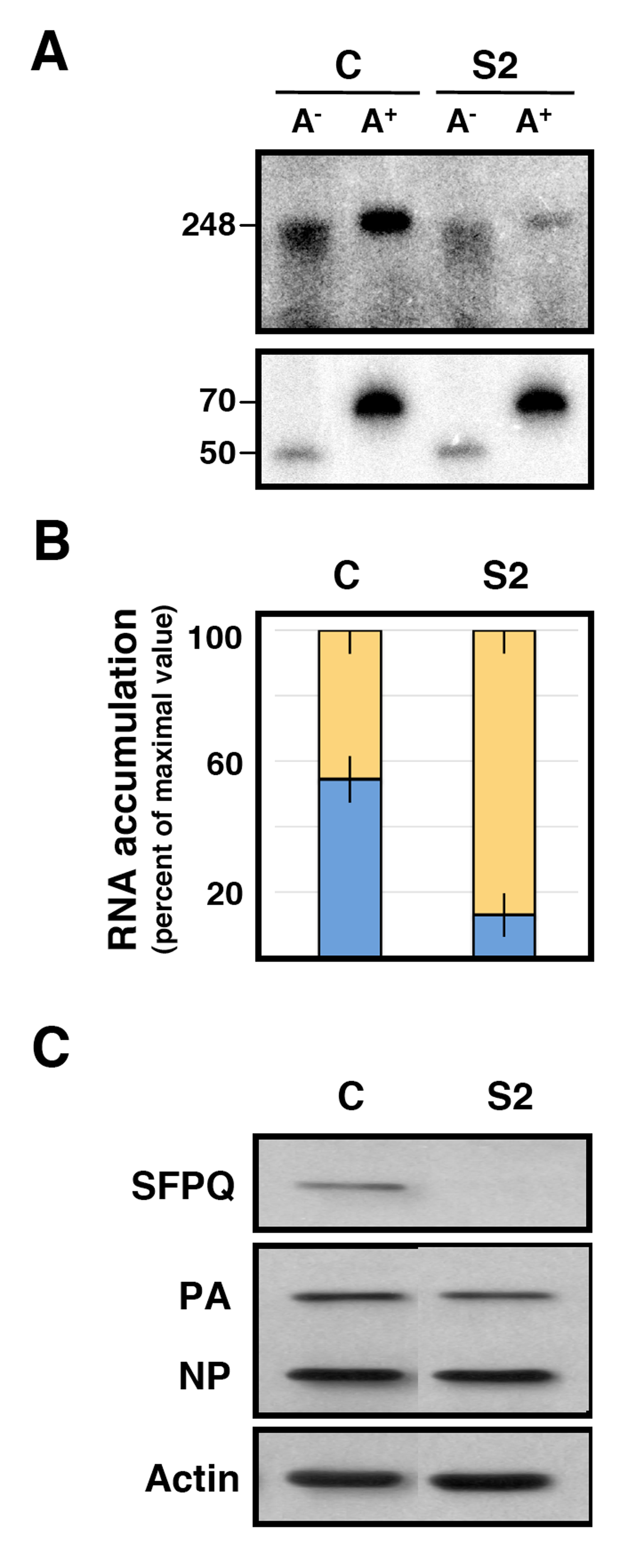

Supplement: Figure S2 — Dependence of SFPQ/PSF for the in vitro polyadenylation of transcripts. Recombinant RNPs were generated in control-silenced (C) or SFPQ/PSF-silenced (S2) HEK293T cells and in vitro transcription was performed as indicated in the legend to Figures 11 and 12. The transcripts were separated into poly A+ (blue) and poly A− (yellow) fractions, using a non-polyadenylated oligonucleotide (50 nt) and a polyadenylated oligonucleotide (70 nt) as recovery probes, and the fractionated transcripts were analysed by electrophoresis on denaturing polyacrylamide gels. (A) Representative results of two independent experiments. The positions of a 248 nt marker identical to the clone 23 genome, as well as the recovery probes are indicated to the left. (B) Quantification of the proportion of the poly A+ and poly A− transcripts after normalisation for recovery. The data represent averages and ranges of two experiments. (C) The silencing of SFPQ was controlled by Western-blot, using actin as loading control. The proper reconstitution of recombinant RNPs was ascertained by Western-blot with antibodies specific for PA and NP. (TIF) [file ppat.1002397.s002.tif]
