# Supplementary material for: The Splicing Factor Proline-Glutamine Rich (SFPQ/PSF) Is Involved in Influenza Virus Transcription
Source: PLoS Pathog. 2011 Nov 17;7(11):e1002397. doi: 10.1371/journal.ppat.1002397 (PMC3219729; doi:10.1371/journal.ppat.1002397)
Supplement: Table S1 — Oligonucleotides used as probes for detection of viral RNAs. The Table shows the sequences of the oligonucleotides used as probes for hybridisation to vRNAs, mRNAs and cRNAs, corresponding to the M, NP and NS segments as indicated. The length of the probes and the concentration of formamide used during the hybridisation reactions are also indicated. Likewise, the sequences used for amplification of NS1 mRNA (NS1 intron), NS2 mRNA (NS2 border) and total NS mRNAs (Total NS) are indicated, as well as the corresponding Taqman probes. (PDF) [file ppat.1002397.s003.pdf]

RNA type	Target segment	Sequence (5'- 3')	Length (nt)	Formamide (%)
vRNA	M	AGATGCAACGATTCAAGTGACCCTCTTGTT	30	41,1
	NP	AAAACCAGAAGAAGTGTCCTTCCGTGGGCG	30	47,4
	NS	AGGACTTGAATGGAATGATAACACAGTTCGA	31	39,4
mRNA	M	TTTTTTTTTTTTTTTTTTTTTACTCCAGTTCAATG	36	30,4
	NP	TTTTTTTTTTTTTTTTTTTTCCTTAATTGTCGTAC	34	28,7
	NS	TTTTTTTTTTTTTTTTTTTTTATCATTAAATAAGCT	37	25,6

RNA type	Target segment	Sequence (5'- 3')	Length (nt)	Formamide (%)
**cRNA**	**M**	AGTAGAAACAAGGTAGTTTTTTACTCCAGT	30	34,9
	**NP**	AGTAGAAACAAGGGTATTTTTCCTTAATTG	30	32,7
	**NS**	AGTAGAAACAAGGGTGTTTTTTATCATTAA	30	30,6

Target gene	Type	Sequence (5'- 3')	Fragment size (bp)
NS1 intron	**Forward**	CCATGTTGGAAAGCAGATAGTAGAGA	105
	**Reverse**	ATGTCAGTTATGTATCGCGAAGCA	
	**Taqman probe Forward**	FAM-ATGAGGCGCTTACAATGA-NFQ	
NS2 border	**Forward**	GCAGGGTGACAAAGACATAATGGAT	93
	**Reverse**	GATGAGGACCCCAATTGCATTTT	
	**Taqman probe Reverse**	FAM-TAGTATGTCCTGAAAACTTG-NFQ	
Total NS	**Forward**	CATCGGAGGACTTGAATGGAATGAT	92
	**Reverse**	GTCCCCCATTCTCATTACTGCTT	
	**Taqman probe Forward**	FAM-AAACTCTACAGAGATTCGC-NFQ	
